# Diabetic retinopathy is a prognostic factor for progression of chronic kidney disease in the patients with type 2 diabetes mellitus

**DOI:** 10.1371/journal.pone.0220506

**Published:** 2019-07-29

**Authors:** Hayne Cho Park, Young-Ki Lee, AJin Cho, Chae hoon Han, Jung-Woo Noh, Young Joo Shin, So Hyun Bae, Hakyoung Kim

**Affiliations:** 1 Department of Internal Medicine, Kangnam Sacred Heart Hospital, Seoul, Korea; 2 Hallym University Kidney Research Institute, Seoul, Korea; 3 Department of Ophthalmology, Kangnam Sacred Heart Hospital, Seoul, Korea; Kaohsiung Medical University Hospital, TAIWAN

## Abstract

Since both retinopathy and nephropathy are major diabetic microvascular complications, we investigated whether severity of diabetic retinopathy (DR) has adverse effects on renal function and albuminuria in the patients with type 2 diabetes mellitus (DM). We screened 2,197 adult patients with type 2 DM who had undergone fundus exam between August 2006 and February 2014. Among them, 1,592 subjects with available serial renal function and albuminuria measurement were included in the analysis. DR status was classified as no DR, non-proliferative DR (NPDR), and proliferative DR (PDR). The risk of CKD progression was assessed according to DR severity. A total of 384 (24.1%) had NPDR and 202 (12.7%) had PDR at either eye. The mean follow-up period was 5.6±2.1 years. DR was associated with lower body mass index, lower plasma hemoglobin, lower serum albumin level, longer duration of DM, poorer control of blood sugar, lower estimated glomerular filtration rate (eGFR), and greater amount of albuminuria. Interestingly, baseline DR severity was associated with faster renal function decline and greater albuminuria progression. In multivariate analysis, NPDR had 2.9 times and PDR had 16.6 times higher risk for CKD progression. Our findings showed that baseline DR severity is a prognostic factor for future CKD progression in type 2 DM patients. Therefore, clinicians must evaluate DR severity at the first visit and closely monitor renal function and albuminuria in the subjects with severe DR.

## Introduction

Both diabetic retinopathy (DR) and nephropathy are typical microvascular complications of diabetes mellitus (DM). It is well known that the prevalence of chronic kidney disease (CKD) and DR increases proportionally to the disease duration in type 2 DM [1, 2]. CKD and DR also share common risk factors such as smoking, poor glycemic control, systolic hypertension, or dyslipidemia [1, 3, 4]. Since both CKD and DR reflect similar pathogenesis and microvascular lesions, it is reasonable to assume that development of DR may predict development and progression of CKD.

However, the association between DR and CKD has not been well established in type 2 DM, and a few studies found that their association in type 2 DM patients is much weaker than that in type 1 DM patients [5, 6]. In addition, the role of DR as a predictor for development and progression of CKD is still controversial. The Microalbuminuria Collaborative Study Group found that DR was not an independent predictor of albuminuria [7], but other researchers found that the presence and severity of DR are still indicators for the risk of developing proteinuria [2, 8].

Recently, a few prospective cohort studies suggested that the presence of retinopathy is related to the development and progression of renal diseases in both diabetic and non-diabetic CKD patients [9–13]. However, there was no study evaluating the effect of DR severity upon CKD progression in type 2 DM patients. Therefore, we aimed to assess the value of DR severity to predict renal dysfunction and albuminuria progression in type 2 DM patients.

## Materials and methods

**Fig 1:**
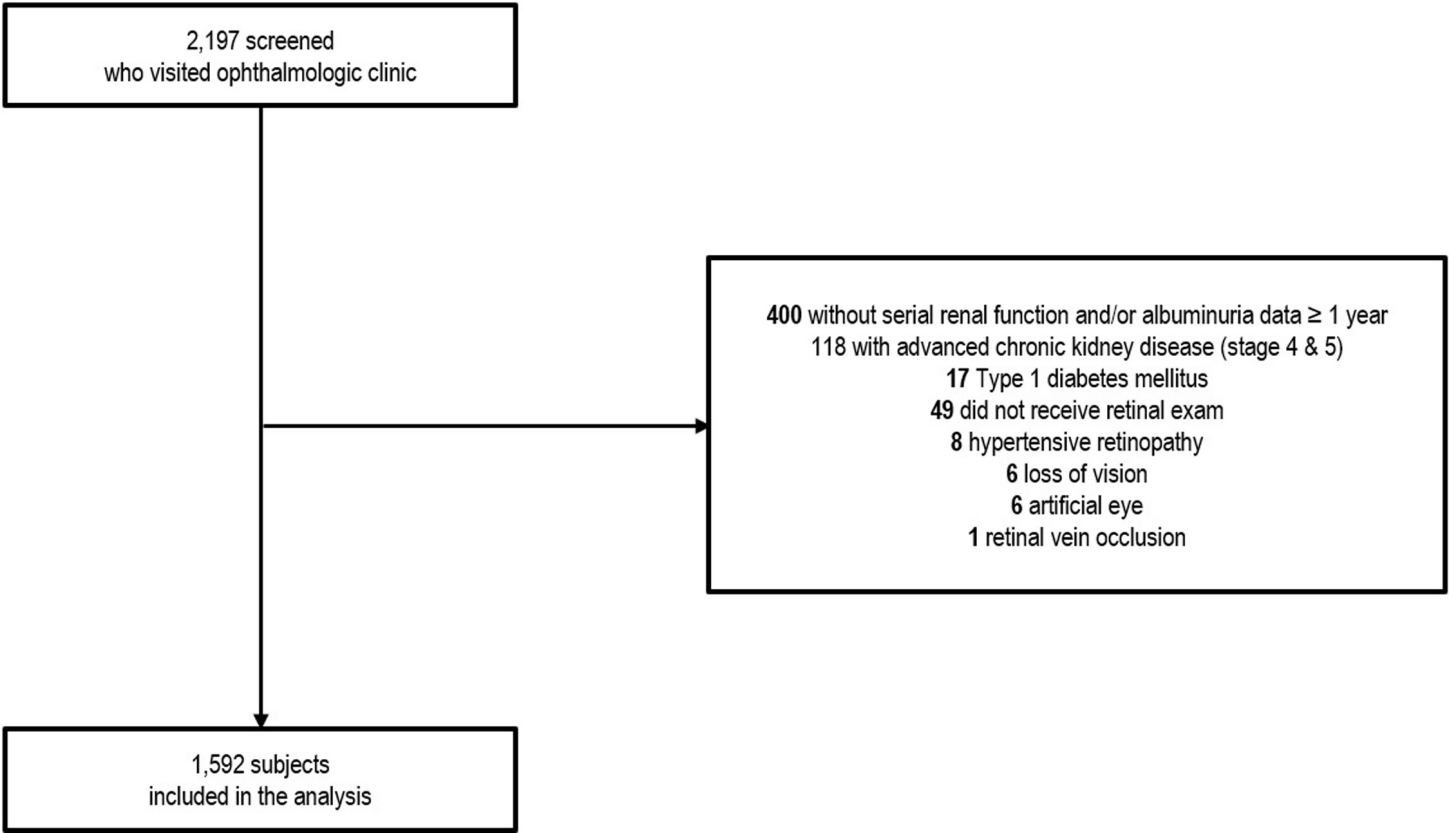
Description of study participants. A total of 2,197 adult DM patients who visited ophthalmologic clinic at Kangnam Sacred Heart Hospital was screened. Among them, 17 patients were type 1 DM, 49 did not received retinal exam, 8 was diagnosed as hypertensive retinopathy, 6 already was blind, 6 had artificial eyes, and 1 had retinal vein occlusion. Another 400 patients were not available for serial renal function data over 1 year and 118 subjects were already in the advanced stage of chronic kidney disease (stage 4 and 5). Therefore, after excluding 605 patients, a total of 1,592 subjects were included in the final analysis for renal outcome.

Medical records from a total of 2,197 adult patients with type 2 DM who visited ophthalmology clinic in Kangnam Sacred Heart Hospital from August 2006 to February 2014 were screened for enrollment. Type 2 DM was diagnosed when the patient is older than 20 years old and presents with one of the followings according to World Health Organization diabetes diagnostic criteria: 1) fasting blood glucose ≥ 6.99 mmol/L, 2) 2-hour plasma glucose level after 75 g oral glucose tolerance test ≥ 11.1 mmol/L, 3) random plasma glucose level ≥ 11.1 mmol/L with diabetic symptoms, 4) glycated hemoglobin (HbA1c) ≥ 48 mmol/mol (6.5%)[14]. Among 2,197 patients, 547 patients were excluded from the analysis due to following reasons: 400 with no serial follow-up data over 1 year (272 with single eGFR measurement, 128 with eGFR follow-up measurement within a year (mean follow-up duration 199.4±103.3 days), 118 with advanced CKD (CKD stage 4 and 5), 17 type 1 DM, 49 without retinal exams, 8 hypertensive retinopathy, 6 loss of vision, 6 artificial eyes, and 1 retinal vein occlusion (Fig 1).

CKD was defined by Kidney Disease: Improving Global Outcomes (KDIGO) clinical practice guideline based on estimated glomerular filtration rate (eGFR) and albuminuria [15]. The eGFR was calculated by CKD-EPI creatinine equation [16]. Serum creatinine was measured by IDMS-traceable method [17]. Annual eGFR decline rate was calculated from the difference of eGFR between recent follow-up and the initial visit divided by follow-up years. The degree of albuminuria was measured by random urine albumin-to-creatinine ratio (UACR). The CKD progression was defined based on one or more of the following: 1) decline in GFR category (≥90 [G1], 60–89 [G2], 45–59 [G3a], 30–44 [G3b], 15–29 [G4], <15 [G5] mL/min/1.73m^2^) accompanied by a 25% or greater drop in eGFR from baseline, 2) sustained declined in eGFR of more than 5 mL/min/1.73m^2^/year [18]. Albuminuria progression was defined by one or more step progression in albuminuria (normo-albuminuria (UACR < 30mg/g) to micro-albuminuria (30mg/g ≤ UACR < 300 mg/g) or macro-albuminuria (UACR ≥ 300 mg/g), micro-albuminuria to macro-albuminuria) during follow-up.

The status of DR was evaluated by slit-lamp examination, indirect ophthalmoscopy and/or fluorescein angiography by ophthalmologists. To evaluate the effect of DR severity upon CKD progression, retinopathy was classified into following categories; no DR, non-proliferative DR (NPDR), and proliferative DR (PDR) [19]. Each eye was given a DR grade and the final DR severity was determined by the result of more severe eye. The progression of DR was defined by more than 1 step progression in DR grade of either eye or the development of PDR requiring photocoagulation or vitrectomy [20].

The following data were obtained from each patient: age, gender, duration of DM, use of angiotensin converting enzyme inhibitor (ACEi) or angiotensin receptor blocker (ARB) at initial visit, body mass index (BMI) at initial visit, serial measurements of serum creatinine, eGFR, UACR, DR status at the initial diagnosis and follow-up, other laboratory findings including plasma hemoglobin, serum albumin, total cholesterol, and HbA1c. The study was approved by the Institutional Review Board (2018-01-030). The written informed consent was waivered due to retrospective nature of the study.

Statistical analysis was conducted using the SPSS software version 20.0 (SPSS, Inc., Chicago, Ill., USA). For descriptive analysis, data were represented either as mean ± standard error or relative frequencies. For normally distributed variables, the Student t-test and one-way ANOVA were used for comparisons. Binary logistic regression analysis was used to evaluate the risk factors for CKD progression. We performed univariate analysis to find risk factors for CKD progression. To seek independent association between each risk factor and CKD progression, we performed multivariate analysis by entering all significant risk factors for CKD progression. The P-value <0.05 was considered statistically significant.

## Results

### Baseline characteristics of the subjects according to the status of diabetic retinopathy

A total of 1,592 patients were included in the analysis (Table 1). Mean age was 57.9 ± 11.2 years old, and female was slightly predominant (n = 841, 52.8%). The mean follow-up period was 5.6±2.1 years. A total of 586 (36.8%) patients had DR at the initial visit. Among them, 384 (24.1%) had NPDR and 202 (12.7%) had PDR at either eye. Compared to the patients without DR, the patients with NPDR and PDR had lower BMI (24.6±3.5 and 23.5±3.4 vs. 25.4±3.6 kg/m^2^, p<0.001), longer duration of DM (11.9±8.0 and 12.0±8.2 vs. 6.7±6.3 years, p<0.001), higher level of HbA1c (8.3±1.8 and 8.5±2.1 vs. 7.5±1.7%, p<0.001), lower hemoglobin level (13.0±1.8 and 12.3±1.8 vs. 13.5±1.6 g/dL, p<0.001) and lower serum albumin level (4.3±0.5 and 4.2±0.6 vs. 4.4±0.4 g/dL, p<0.001). In addition, patients with NPDR and PDR showed decreased eGFR (76.1±19.6 and 72.4±22.3 vs. 81.5±18.5 mL/min/1.73m^2^, p<0.001) and increased amount of albuminuria (median 12.6 and 23.1 vs. 37.0 mg/g, p<0.001) compared to those without DR.

**Table 1:** Baseline characteristics of the subjects classified by diabetic retinopathy.

Variables	No DR (n = 1,006)	NPDR (n = 384)	PDR (n = 202)	p-value
Age (years)	57.5±11.2	59.3±10.9	57.3±11.2	0.016
Male (%)	470 (46.7)	175 (45.6)	106 (52.5)	0.252
Hypertension (%)	485 (50.2)	201 (54.6)	87 (43.5)	0.04
BMI (kg/m^2^)	25.4±3.6	24.6±3.5	23.5±3.4	<0.001
DM duration (years)	6.7±6.3	11.9±8.0	12.0±8.2	<0.001
ACEi or ARB (%)	471 (46.8)	202 (52.6)	97 (48.0)	0.154
sBP (mmHg)	128.8±18.7	131.8±20.2	133.2±22.6	0.026
dBP (mmHg)	76.9±12.7	78.9±13.2	79.0±15.1	0.057
HbA1c (%)	7.5±1.7	8.3±1.8	8.5±2.1	<0.001
FPG (mg/dL)	143.2±59.9	153.6±68.0	171.4±96.3	<0.001
Creatinine (mg/dL)	0.93±0.23	0.99±0.27	1.08±0.34	<0.001
eGFR (mL/min/1.73m^2^)	81.5±18.5	76.1±19.6	72.4±22.3	<0.001
UACR (mg/g)	12.6 [7.0, 24.5]	23.1 [10.6, 98.9]	37.0 [16.5, 235.5]	<0.001
Hemoglobin (g/dL)	13.5±1.6	13.0±1.8	12.3±1.8	<0.001
Total cholesterol (mg/dL)	167.1±35.8	166.9±41.2	168.4±41.9	0.892
Albumin (g/dL)	4.4±0.4	4.3±0.5	4.2±0.6	<0.001

DR, diabetic retinopathy; NPDR, non-proliferative diabetic retinopathy; PDR, proliferative diabetic retinopathy; BMI, body mass index; DM, diabetes mellitus; ACEi, angiotensin converting enzyme inhibitor; ARB, angiotensin receptor blocker; sBP, systolic blood pressure; dBP, diastolic blood pressure; HbA1c, hemoglobin A1c; FPG, fasting plasma glucose; eGFR, estimated glomerular filtration rate; UACR, urinary albumin-to-creatinine ratio

### Severity of diabetic retinopathy affects the rate of renal function decline

**Fig 2:**
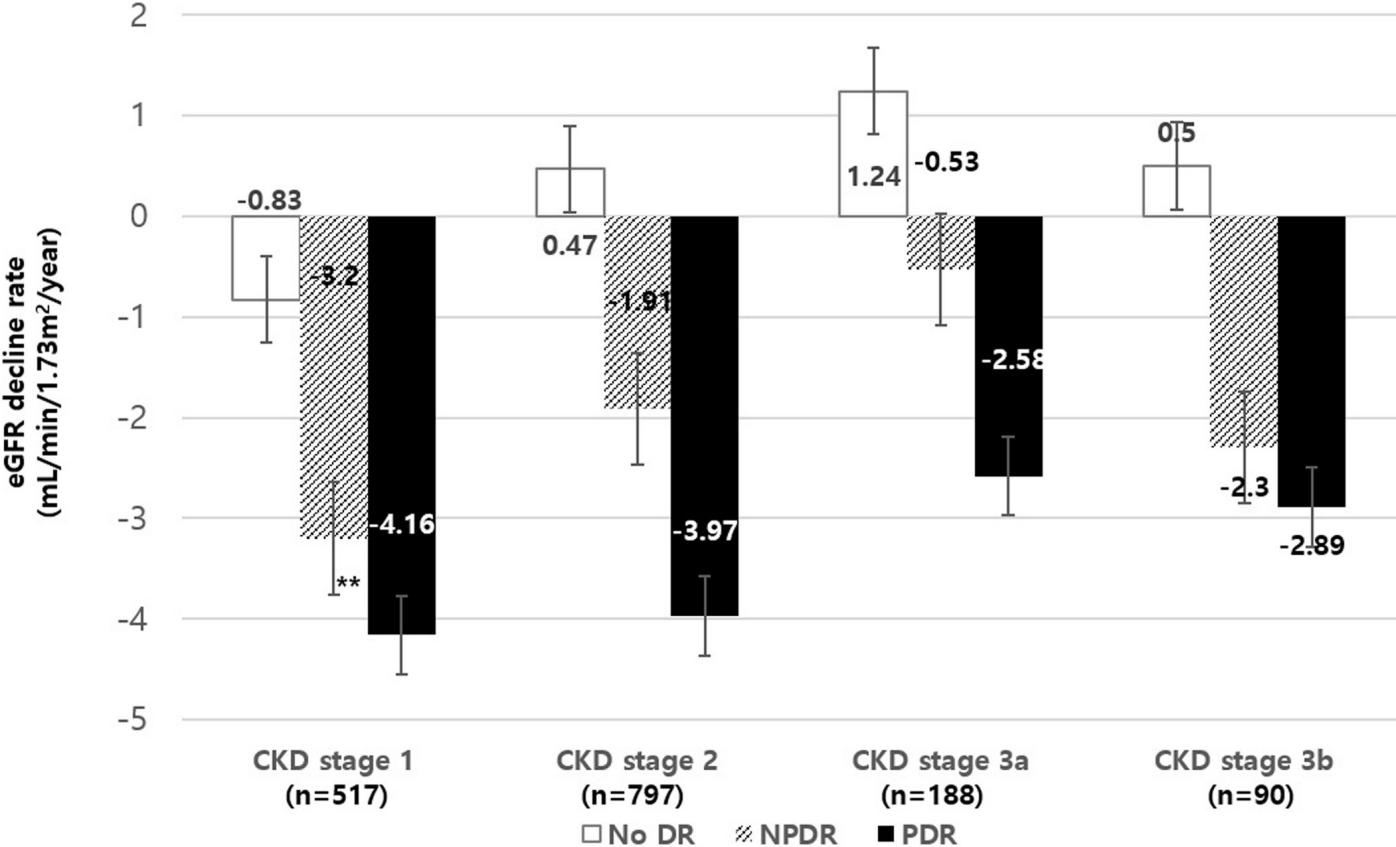
Annual renal function decline rate according to initial diabetic retinopathy status. To exclude the effect of baseline renal function upon future renal function decline rate, we performed subgroup analysis according to baseline CKD stages based on eGFR. In CKD stage 1 (n = 517), patients with NPDR and PDR at baseline showed faster decline of renal function compared to those without DR (-3.2±6.44 and -4.16±5.43 vs. -0.83±3.48 mL/min/1.73m^2^/year, p<0.001). The patients with NPDR and PDR with baseline CKD stages 2 (n = 797), 3a (n = 188), and 3b (n = 90) showed decline in renal function during follow up (-1.91±5.71, -0.53±4.25 and -2.3±3.34 mL/min/1.73m^2^ per year for NPDR and -3.97±6.08, -2.58±5.71 and -2.89±9.19 mL/min/1.73m^2^ per year for PDR) while those without DR showed preserved renal function during follow-up (0.47±3.22, 1.24±3.91 and 0.5±4.34 mL/min/1.73m^2^ per year, p<0.001). eGFR, estimated glomerular filtration rate; CKD, chronic kidney disease; DR, diabetic retinopathy; NPDR, non-proliferative diabetic retinopathy; PDR, proliferative diabetic retinopathy.

To assess whether DR severity affects subsequent renal function decline rate, we compared annual decline rate of eGFR among subjects with different DR severity. Since baseline renal function is a strong predictor of future eGFR decline, we divided subjects into groups based on baseline CKD stages according to GFR categories (Fig 2). Among the subjects with baseline CKD stage 1 (n = 517), patients with NPDR (n = 102) and PDR (n = 51) showed faster eGFR decline rate (-3.2±6.44 and -4.16±5.43 mL/min/1.73m^2^ per year) compared to those without DR (n = 364, -0.83±3.48 mL/min/1.73m^2^ per year, p<0.001). The subjects in CKD stage 2 (n = 797) at baseline showed preserved renal function among no DR subjects (0.47±3.22 mL/min/1.73m^2^ per year) while rapid progression in NPDR and PDR (-1.91±5.71 and -3.97±6.08 mL/min/1.73m^2^ per year) patients (p<0.001). The results were similar for those in CKD stage 3a (n = 188) and 3b (n = 90) showing preserved renal function in no DR subjects (1.24±3.91 and 0.5±4.34 mL/min/1.73m^2^ per year) while rapid progression in NPDR (-0.53±4.25 and -2.3±3.34 mL/min/1.73m^2^ per year) and PDR (-2.58±5.71 and -2.89±9.19 mL/min/1.73m^2^ per year) patients (p<0.001).

### Diabetic retinopathy is a risk factor for CKD progression

**Fig 3:**
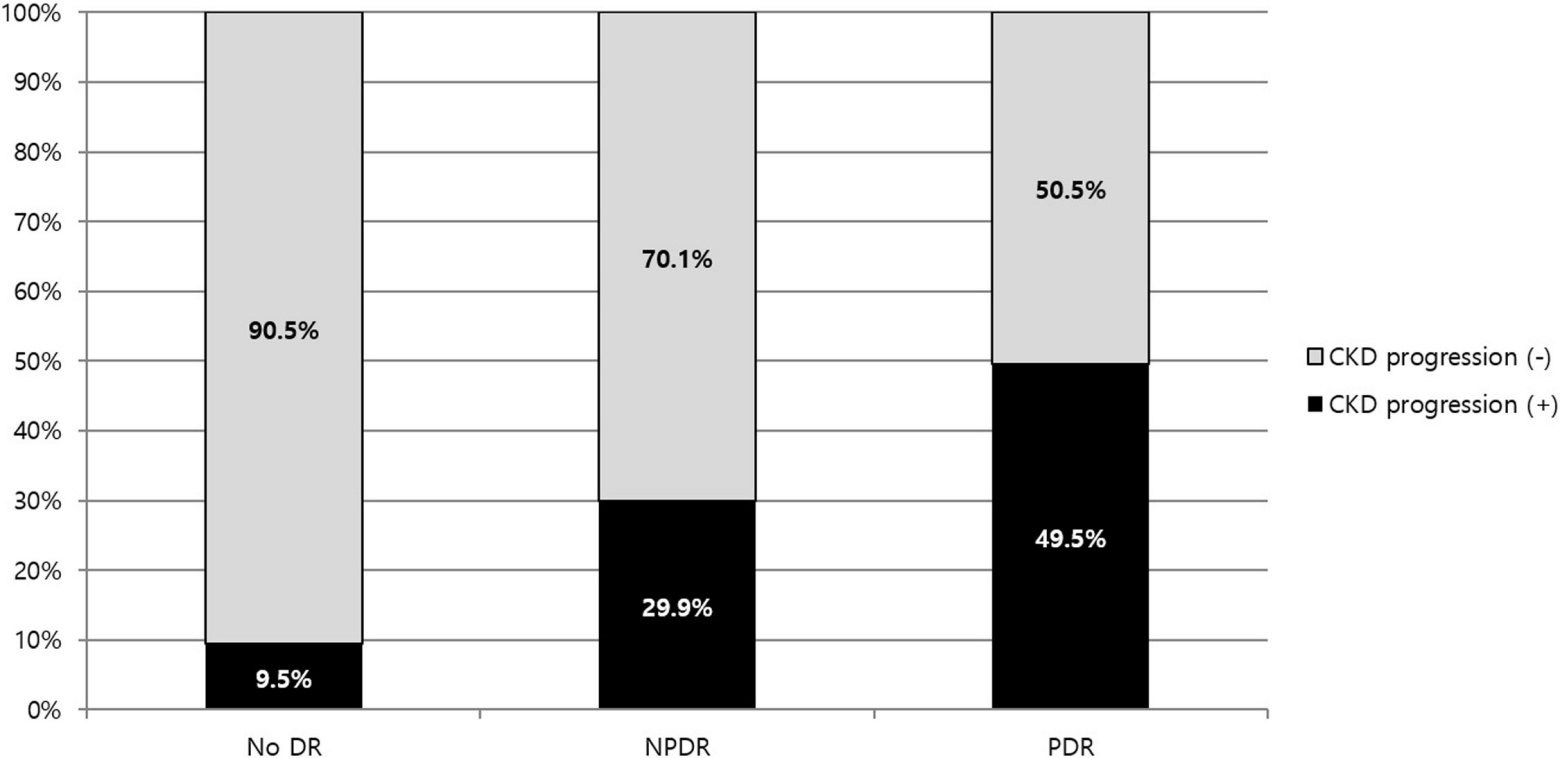
Proportion of the subjects with CKD progression according to initial diabetic retinopathy status. CKD progression was defined by one or more of the following: 1) decline in GFR category (≥90 [G1], 60–89 [G2], 45–59 [G3a], 30–44 [G3b], 15–29 [G4], <15 [G5] mL/min/1.73m^2^) accompanied by a 25% or greater drop in eGFR from baseline, 2) sustained decline in eGFR of more than 5 mL/min/1.73m^2^/year. The proportion of the subjects with CKD progression increased as DR severity increased (9.5 vs. 29.9 vs. 49.5%, p<0.001). GFR, glomerular filtration rate; DR, diabetic retinopathy; NPDR, non-proliferative diabetic retinopathy; PDR, proliferative diabetic retinopathy.

To evaluate the effect of DR severity upon CKD progression, 1,592 subjects were further divided into group of CKD progression (n = 311) and non-progression (n = 1,281, Table 2). The subjects with CKD progression showed lower BMI (24.4±3.7 vs. 25.2±3.6 kg/m^2^, p = 0.014), longer duration of DM (11.2±8.2 vs. 8.0±7.1 years, p<0.001), more frequent use of ACEi or ARB (55% vs. 46.8%, p = 0.009) and higher HbA1c (8.6±2.2 vs. 7.6±1.7%, p<0.001). They also showed lower baseline eGFR (73.0±22.6 vs. 80.5±18.5 mL/min/1.73m^2^, p<0.001), greater amount of albuminuria (median 13.5 vs. 36.7 mg/g, p = 0.001), lower plasma hemoglobin (12.5±2.0 vs. 13.4±1.6 g/dL, p<0.001), and lower serum albumin level (4.1±0.6 vs. 4.5±0.4 g/dL, p<0.001). The subjects with CKD progression also showed larger proportion of NPDR and PDR (37.0% and 32.2%) compared to those without CKD progression (21.0% and 8.0%, p<0.001). The proportion of those with CKD progression increased as DR severity increased (9.5% vs. 29.9% vs. 49.5%, p<0.001, Fig 3). The group with CKD progression showed higher proportion of DR progression (25.5% vs. 16.2%, p = 0.003).

**Table 2:** Risk factors associated with CKD progression.

Parameters	CKD progression		P- value
	(-) (n = 1,281)	(+) (n = 311)	
Age (years)	57.7±11.0	58.6±12.0	0.204
Male (%)	46.3%	50.8%	0.164
Hypertension (%)	50.3%	46.7%	0.275
BMI (kg/m^2^)	25.2±3.6	24.4±3.7	0.014
DM duration (years)	8.0±7.1	11.2±8.2	<0.001
ACEi or ARB (%)	599 (46.8)	171 (55.0)	0.009
HbA1c (%)	7.6±1.7	8.6±2.2	<0.001
Cr at baseline (mg/dL)	0.94±0.23	1.06±0.33	<0.001
eGFR at baseline (mL/min/1.73m^2^)	80.5±18.5	73.0±22.6	<0.001
UACR at baseline (mg/g)	13.5 [7.4, 27.1]	36.7 [13.3, 226.3]	0.001
Hemoglobin (g/dL)	13.4±1.6	12.5±2.0	<0.001
Total cholesterol (mg/dL)	166.3±35.0	170.9±48.0	0.114
Albumin (g/dL)	4.5±0.4	4.1±0.6	<0.001
DR at baseline (%) No DR NPDR PDR	71.0% 21.0% 8.0%	30.9% 37.0% 32.2%	<0.001
DR progression (%)	16.2%	25.5%	0.003

eGFR, estimated glomerular filtration rate; BMI, body mass index; DM, diabetes mellitus; ACEi, angiotensin converting enzyme inhibitor; ARB, angiotensin receptor blocker; HbA1c, hemoglobin A1c; UACR, urinary albumin-to-creatinine ratio; DR, diabetic retinopathy; NPDR, non-proliferative diabetic retinopathy; PDR, proliferative diabetic retinopathy

### Diabetic retinopathy is a risk factor for albuminuria progression

**Fig 4:**
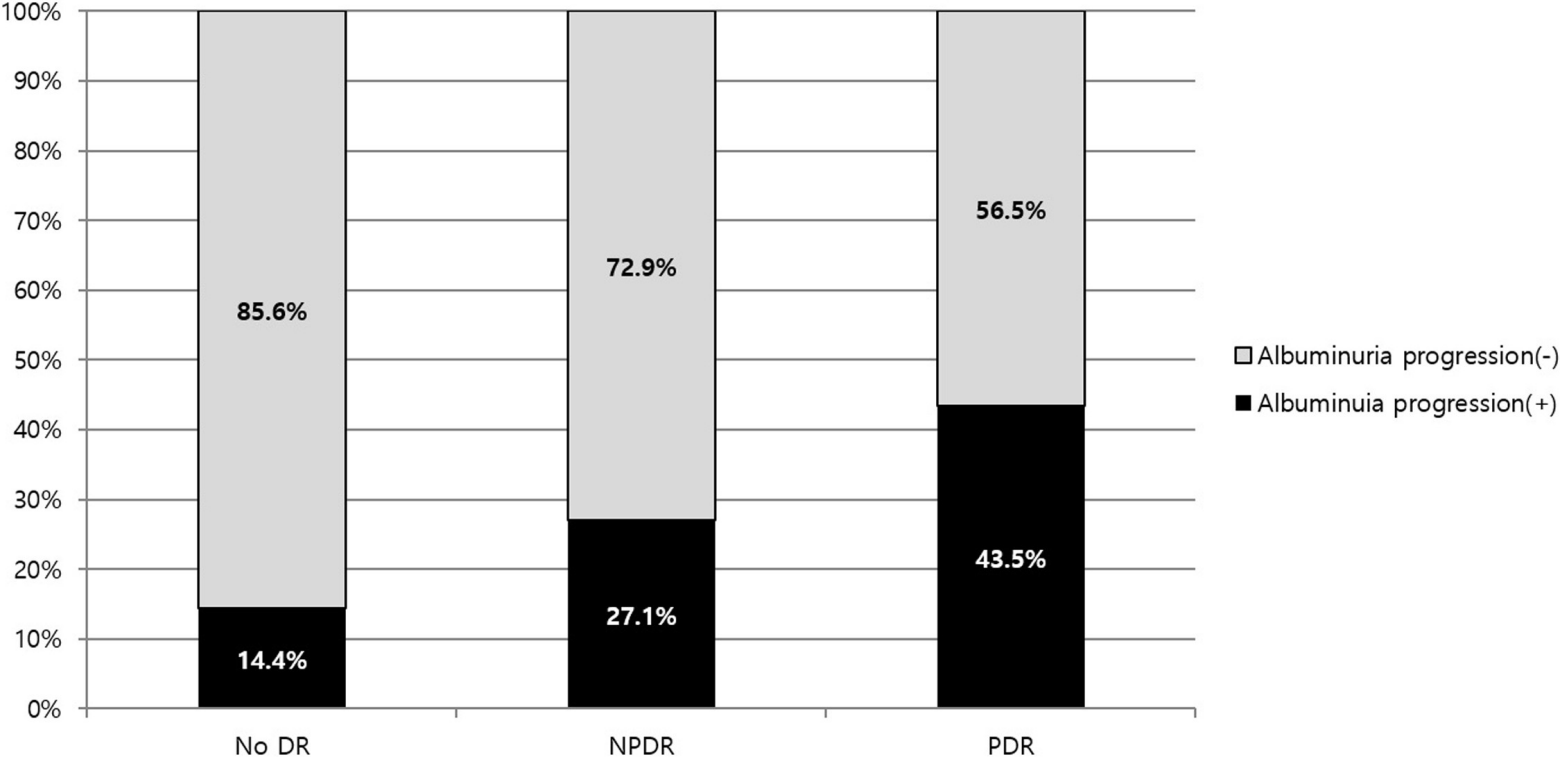
Proportion of the subjects with albuminuria progression according to initial diabetic retinopathy status. Albuminuria progression was defined by one or more step progression in albuminuria (normo-albuminuria (UACR < 30mg/g) to micro-albuminuria (30mg/g ≤ UACR < 300 mg/g) or macro-albuminuria (UACR ≥ 300 mg/g), micro-albuminuria to macro-albuminuria) during follow-up. The proportion of the subjects with albuminuria progression increased as DR severity increased (14.4% vs. 27.1% vs. 43.5%, p<0.001). DR, diabetic retinopathy; NPDR, non-proliferative diabetic retinopathy; PDR, proliferative diabetic retinopathy.

To evaluate the effect of DR severity upon albuminuria progression, a total of 948 subjects with serial albuminuria data over 1 year were included in the analysis (Table 3). A total of 644 subjects were excluded from the analysis for the following reasons: 546 without initial albuminuria data, 61 with initial macroalbuminuria (UACR≥300mg/g), and 37 without serial albuminuria data. Among 948 subjects, 180 patients showed albuminuria progression while 768 patients showed stable amount of albuminuria. The group with albuminuria progression showed longer duration of DM (9.2±7.4 vs. 7.7±6.9 years, p = 0.008), more frequent use of ACEi or ARB (67.8% vs. 50.9%, p<0.001), higher HbA1c (8.2±2.2 vs. 7.5±1.7%, p<0.001) and lower baseline eGFR (76.7±20.1 vs. 82.3±17.5 mL/min/1.73m^2^, p = 0.001) and higher amount of baseline UACR (median 13.6 vs. 18.5 mg/g, p = 0.014). As same as those with CKD progression, the group with albuminuria progression showed larger proportion of NPDR and PDR compared to those without progression (30.0% and 15.0% vs. 18.8% and 4.5%, p<0.001). The proportion of the subjects with albuminuria progression increased as DR severity increased (14.4% vs. 27.1% vs. 43.5%, p<0.001, Fig 4). However, proportion of DR progression during follow-up was not different between two groups (18.8% vs. 13.9%, p = 0.148).

**Table 3:** Risk factors associated with albuminuria progression.

Parameters	Albuminuria progression		P- value
	(-) (n = 768)	(+) (n = 180)	
Age (years)	56.5±10.6	57.4±12.0	0.328
Male (%)	47.4%	40.0%	0.081
Hypertension (%)	46.9%	52.3%	0.206
BMI (kg/m^2^)	25.1±3.5	25.2±3.6	0.75
DM duration (years)	7.7±6.9	9.2±7.4	0.008
ACEi or ARB (%)	391 (50.9)	122 (67.8)	<0.001
HbA1c (%)	7.5±1.7	8.2±2.2	<0.001
Cr at baseline (mg/dL)	0.92±0.21	0.98±0.26	0.006
eGFR at baseline (mL/min/1.73m^2^)	82.3±17.5	76.7±20.1	0.001
UACR at baseline (mg/g)	13.6 [7.4, 27.4]	18.5 [10.1, 28.5]	0.014
Hemoglobin (g/dL)	13.5±1.7	13.3±1.5	0.127
Total cholesterol (mg/dL)	163.8±34.0	164.0±35.2	0.941
Albumin (g/dL)	4.5±0.4	4.4±0.4	0.198
DR at baseline (%) No DR NPDR PDR	76.6% 18.9% 4.6%	55.0% 30.0% 15.0%	<0.001
DR progression (%)	13.9%	18.8%	0.148

BMI, body mass index; DM, diabetes mellitus; ACEi, angiotensin converting enzyme inhibitor; ARB, angiotensin receptor blocker; HbA1c, hemoglobin A1c; eGFR, estimated glomerular filtration rate; UACR, urinary albumin-to-creatinine ratio; DR, diabetic retinopathy; NPDR, non-proliferative diabetic retinopathy; PDR, proliferative diabetic retinopathy

### Diabetic retinopathy is a prognostic factor for progression of chronic kidney disease

To assess whether DR severity is a prognostic factor for CKD progression, we performed binary logistic regression analysis. In the univariate analysis, low BMI, longer DM duration, use of ACEi or ARB at the initial visit, higher HbA1c, lower baseline eGFR, higher baseline UACR, lower plasma hemoglobin, lower serum albumin, presence of DR, and presence of DR progression were all related to the CKD progression (Table 4, p<0.05).

**Table 4:** Binary logistic regression analysis for CKD progression.

Parameter	Odds ratio	Confidence interval	p-value
Age, yr	1.007	0.996–1.019	0.204
Male	1.198	0.935–1.535	0.153
HTN	1.157	0.899–1.489	0.258
BMI, kg/m^2^	0.943	0.899–0.988	0.014
DM duration, yr	1.055	1.039–1.072	<0.001
Use of ACEi or ARB	1.391	1.084–1.784	0.009
HbA1c, %	1.308	1.227–1.395	<0.001
Baseline eGFR, mL/min/1.73m^2^	0.981	0.974–0.987	<0.001
Baseline UACR, mg/g	1.002	1.001–1.003	<0.001
Plasma hemoglobin, g/dL	0.743	0.689–0.801	<0.001
Total cholesterol, mg/dL	1.003	1.000–1.006	0.057
Serum albumin, g/dL	0.186	0.14–0.247	<0.001
NPDR (vs. no DR)	4.052	2.993–5.488	<0.001
PDR (vs. no DR)	9.293	6.569–13.146	<0.001
DR progression	1.769	1.230–2.545	0.002

CKD, chronic kidney disease; HTN, hypertension; BMI, body mass index; DM, diabetes mellitus; ACEi, angiotensin converting enzyme inhibitor; ARB, angiotensin receptor blocker; HbA1c, hemoglobin A1c; eGFR, estimated glomerular filtration rate; UACR, urinary albumin-to-creatinine ratio; NPDR, non-proliferative diabetic retinopathy; PDR, proliferative diabetic retinopathy; DR, diabetic retinopathy

When we performed multiple logistic analysis after adjusting for DM duration, use of ACEi or ARB, HbA1c, baseline eGFR, baseline UACR, plasma hemoglobin and serum albumin levels, baseline DR severity and DR progression status, DR severity was independently associated with CKD progression showing that NPDR has 2.9 times and PDR has 16.6 times higher risk for CKD progression compared to no DR group (Table 5, p for trend <0.001). However, DR progression during follow-up period was not an independent risk factor for CKD progression (p = 0.563).

**Table 5:** Multiple logistic regression analysis for prediction of CKD progression.

Parameter	Odds ratio	Confidence interval	p-value
Baseline eGFR <60 mL/min/1.73m^2^	2.068	1.062–4.027	0.033
Baseline UACR ≥30 mg/g	3.314	1.847–5.947	<0.001
Albumin <4.0 g/dL	2.840	1.495–5.394	0.001
NPDR (vs. no DR)	2.910	1.602–5.285	<0.001
PDR (vs. no DR)	16.582	2.431–113.123	0.004
DR progression	1.213	0.631–2.330	0.563
Hemoglobin <10 g/dL	1.849	0.619–5.520	0.271
HbA1c ≥7.0%	1.703	0.901–3.218	0.101
BMI <25 kg/m^2^	1.642	0.942–2.860	0.08
DM duration ≥10years	0.978	0.537–1.780	0.942
Use of ACEi or ARB	1.121	0.625–2.009	0.702

CKD, chronic kidney disease; HbA1c, hemoglobin A1c; NPDR, non-proliferative diabetic retinopathy; PDR, proliferative diabetic retinopathy; DR, diabetic retinopathy; BMI, body mass index; DM, diabetes mellitus; eGFR, estimated glomerular filtration rate; UACR, urinary albumin-to-creatinine ratio

## Discussion

The current study was performed to show the importance of baseline DR severity upon prospective renal function decline and albuminuria progression. Our study showed that DR was associated with longer duration of DM, poorer control of blood sugar, lower eGFR, greater amount of albuminuria, and poorer nutritional indices (serum albumin, BMI, and plasma hemoglobin). Interestingly, baseline DR severity was associated with progressive renal function decline and albuminuria progression. Even after adjusting for other risk factors, NPDR had 2.9 times and PDR had 16.6 times higher risk for CKD progression in type2 DM subjects. On the other hand, DR progression during follow up period did not affect future CKD progression.

It is well known that the association between DR and CKD is strong and the presence of CKD almost always accompany DR [21]. However, this association is known to be weaker in type 2 DM patients [5, 6]. A large amount of cross-sectional studies demonstrated that the presence of DR is associated with concurrent renal dysfunction [9, 12, 13, 22]. However, only a few studies have evaluated causal relationship between DR and CKD. Trevisan et al. demonstrated that the rate of renal function decline was larger with those who have retinopathy and proteinuria compared to those without retinopathy [23]. Another study evaluated the effect of retinopathy upon renal outcome in elderly group and showed that retinopathy even affects faster renal function decline in non-DM subjects [8]. There was also the study evaluating the effect of DR upon renal outcome compared to the non-diabetic hypertensive retinopathy [11]. However, there was no study to evaluate the renal outcome according to DR severity.

Our study is the first large-scale study to evaluate the effect of DR severity upon renal outcome. Previous association studies only described the association of the ‘presence of DR’ with either eGFR decline or proteinuria progression. However, our study is the first study to evaluate the effect of ‘degree of DR’ but not the presence of DR upon CKD progression.

There can be some possible explanation about why DR severity affects renal function deterioration. Firstly, DR and diabetic CKD are both microvascular complication which lead to extravasation and inflammation. Previous study by Matsuyama et al. suggested that pigment epithelium-derived factor, an inhibitor for angiogenesis, is significantly elevated in the type 2 DM patients with DR and CKD, which may indicate microvascular damage [24]. Another study by Yang et al. also suggested that retinal damage marker found in urine proteome reflect renal progression in type 2 DM patients [25]. However, other recent study by McKay et al. showed that mere retinal microvascular parameters including vascular caliber, tortuosity, and fractal dimension cannot predict renal outcome in type 2 DM patients [26]. Moriya et al. suggested that microalbuminuria together with DR result in glomerulosclerosis and renal progression [27]. However, our study suggested that DR severity independently affect renal function deterioration after adjustment for albuminuria status.

Meanwhile, DR progression was not an independent risk factor for further progression of CKD. This may be a part due to missing data. Only 1,422 patients had available follow-up retinal exam more than a year apart and another 170 patients with both PDR at the baseline were excluded from the data analysis. In addition, some subjects were excluded from the analysis due to advanced CKD at baseline (n = 108), macro-albuminuria at baseline (n = 61), and no available albuminuria data (n = 546). Therefore, whether DR progression may reflect the renal outcome should be assessed in the future in a large prospective cohort study.

This study has several limitations. The data in this study was collected retrospectively from a single center. Secondly, we did not collect 24-hour urine samples to quantify the amount of albuminuria. We used albumin-to-creatinine ratio in random urine to quantify the amount of albuminuria. In addition, there were some missing data regarding serial follow up of albuminuria. Thirdly, we neither perform kidney biopsy to see the severity of diabetic nephropathy nor unravel the possible mechanism underlying relationship between DR and CKD. Lastly, this study design limits inferences on causality. These subjects were from the Korean population and were visited in the hospital and the results cannot be generalized to general population and other ethnic groups. Therefore, prospective, cohort-based study should be warranted to reproduce and confirm our study finding.

In conclusion, DR was a prognostic factor for CKD progression in type2 DM patients. Therefore, clinicians must evaluate DR severity at the first visit and closely monitor renal function and albuminuria in the subjects with severe DR.
